# Supplementary material for: Studies on the Relationships between Growth and Gonad Development during First Sexual Maturation of Macrobrachium nipponense and Associated SNPs Screening
Source: Int J Mol Sci. 2024 Jun 27;25(13):7071. doi: 10.3390/ijms25137071 (PMC11241762; doi:10.3390/ijms25137071)
Supplement: Supplementary file 1 [file ijms-25-07071-s001.zip › ijms-3048148-supplementary.pdf]

Table S1

Table S1. Genotype frequency and diversity parameters of SNPs in females

Number	SNPs	Genotype	Genotype Frequency	*Ne*	*Ho*	*He*	*PIC*
1	A+118T	TT	0.80	1.3824	0.7226	0.7774	0.24
		AA	0.13				
		AT	0.07				
2	C+155G	GG	0.80	1.3161	0.7592	0.7408	0.20
		CC	0.07				
		GC	0.13				
3	A+206G	GG	0.81	1.3528	0.7386	0.7614	0.22
		AA	0.11				
		AG	0.08				
4	T+216G	GG	0.67	1.5582	0.6408	0.5592	0.28
		TT	0.13				
		GT	0.20				
5	A+333G	AA	0.19	1.4415	0.6929	0.6071	0.26
		GG	0.81				
6	T+521A	TT	0.30	1.7144	0.5822	0.5178	0.33
		AA	0.70				
7	C+640T	TT	0.32	1.7622	0.5664	0.5336	0.34
		CC	0.68				
8	C+642T	TT	0.71	1.7021	0.5865	0.5135	0.33
		CC	0.29				
9	T+698C	TT	0.30	1.7266	0.5781	0.4219	0.33

		CC	0.70				
10	A+709T	AA	0.53	1.9925	0.5006	0.4981	0.37
		TT	0.47				
11	A+782G	AA	0.30	1.7326	0.5761	0.4239	0.33
		GG	0.69				
		AG	0.01				
12	A+1379C	AA	0.32	1.7963	0.5556	0.4444	0.34
		CC	0.64				
		AC	0.04				
13	C+1698G	GG	0.76	1.5540	0.6426	0.5574	0.29
		CC	0.23				
		GC	0.01				
14	C+1717T	TT	0.64	1.8338	0.5441	0.4559	0.35
		CC	0.34				
		CT	0.02				
15	A+1884C	AA	0.30	1.7446	0.5721	0.5279	0.34
		CC	0.69				
		AC	0.01				
16	A+1885G	AA	0.27	1.6581	0.6021	0.5279	0.32
		GG	0.72				
		AG	0.01				

Notes: *Ne*, effective allele numbers; *Ho*, observed heterozygosity; *He*, expected heterozygosity; HWpval

Table S2

Table S2. Genotype frequency and diversity parameters of SNPs in males

Number	SNPs	Genotype	Genotype Frequency	*Ne*	*Ho*	*He*	*PIC*
1	A+118T	TT	0.80	1.3738	0.7272	0.7728	0.31
		AA	0.12				
		AT	0.08				
2	C+155G	GG	0.78	1.3314	0.7505	0.6495	0.33
		CC	0.07				
		GC	0.15				
3	A+206G	GG	0.81	1.3352	0.7483	0.5517	0.30
		AA	0.10				
		AG	0.09				
4	T+216G	GG	0.68	1.5364	0.6500	0.5500	0.22
		TT	0.12				
		GT	0.19				
5	A+333G	AA	0.17	1.3998	0.7137	0.6863	0.24
		GG	0.83				
6	T+521A	TT	0.28	1.6861	0.5920	0.4080	0.32
		AA	0.72				
7	C+640T	TT	0.29	1.7108	0.5835	0.5165	0.32
		CC	0.71				
8	C+642T	TT	0.71	1.6985	0.5877	0.4123	0.32
		CC	0.29				
9	T+698C	TT	0.28	1.7069	0.5848	0.4152	0.33

		CC	0.72				
10	A+709T	AA	0.54	2.0070	0.4970	0.5030	0.37
		TT	0.46				
11	A+782G	AA	0.26	1.6389	0.6092	0.4008	0.31
		GG	0.74				
12	A+1379C	AA	0.30	1.8127	0.5505	0.4495	0.44
		CC	0.62				
		AC	0.08				
13	C+1698G	GG	0.75	1.6000	0.6240	0.4760	0.30
		CC	0.25				
14	C+1717T	TT	0.59	1.9162	0.5207	0.4793	0.39
		CC	0.39				
		CT	0.02				
15	A+1884C	AA	0.30	1.7446	0.5721	0.4268	0.34
		CC	0.69				
		AC	0.01				
16	A+1885G	AA	0.25	1.6065	0.6215	0.4785	0.30
		GG	0.75				

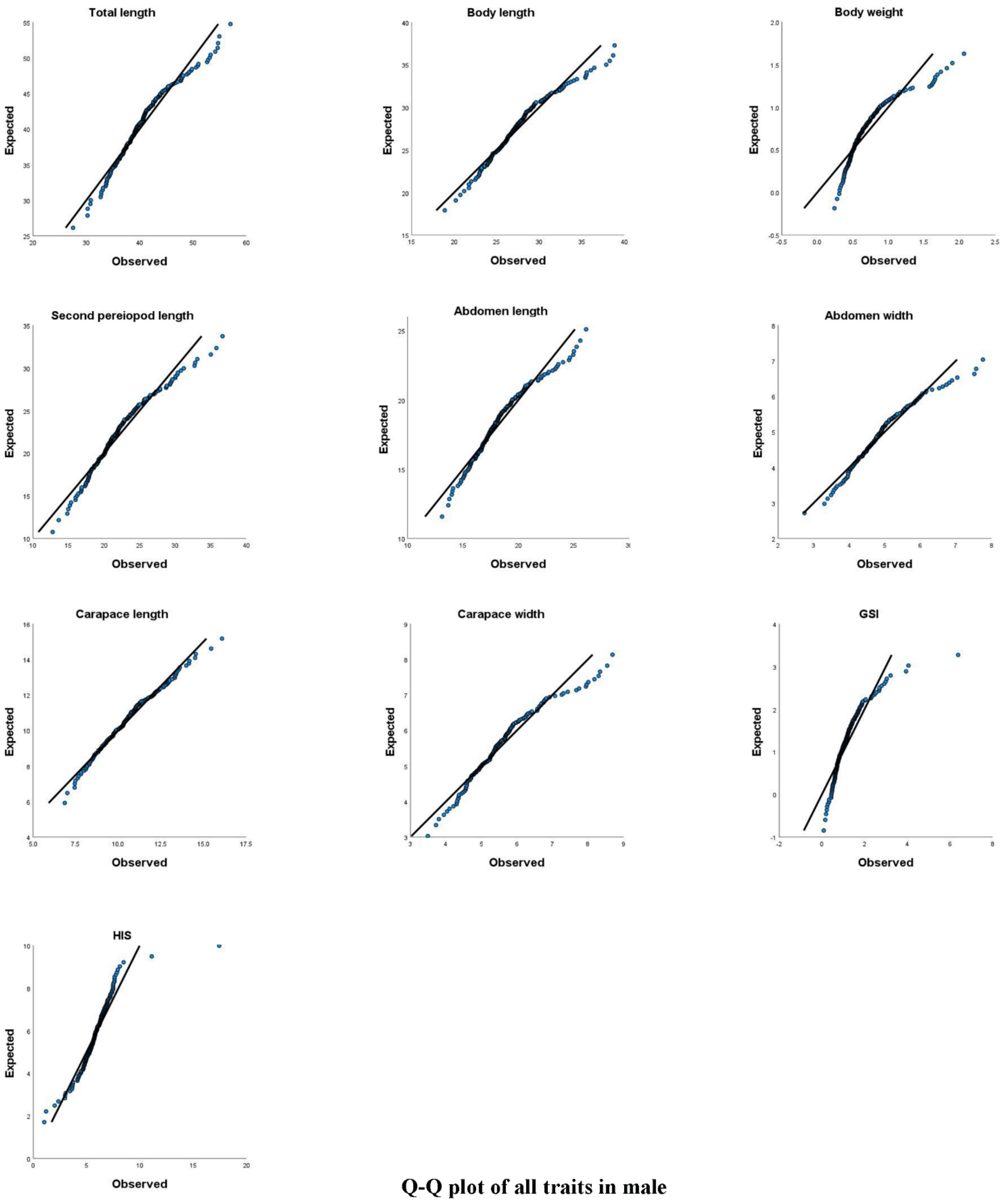

Figure S1. Q-Q plot for all traits in males.

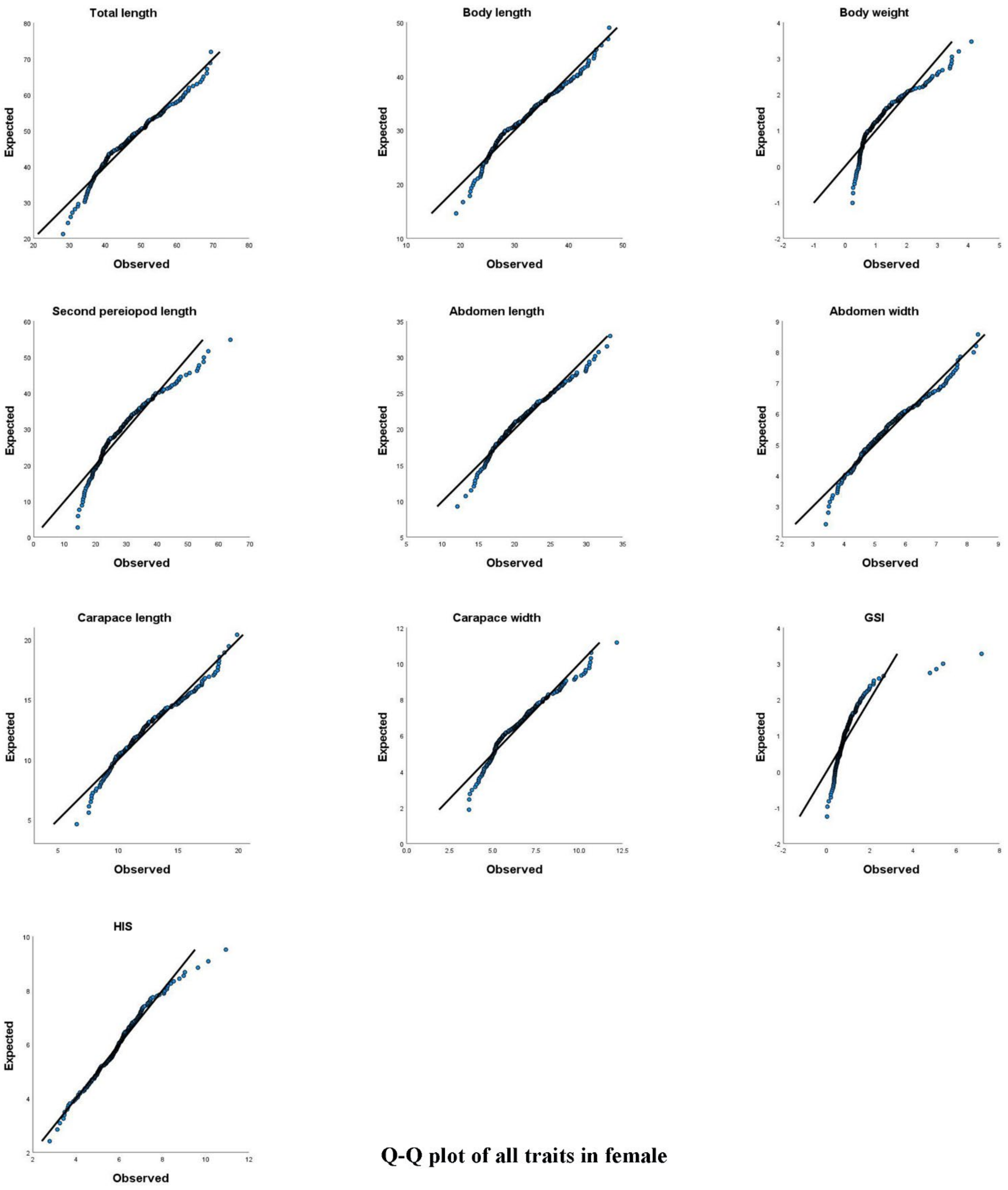

Figure S2. Q-Q plot for all traits in females.
